# Immunoscore and Post-Neoadjuvant Pathological Features in Microsatellite-Stable Locally Advanced Gastric Cancer: A Retrospective Cohort Study

**DOI:** 10.3390/cancers18203329

**Published:** 2026-10-15

**Authors:** Özlem Kutlu, Gürkan Gül, Simge Baran, Asuman Argon, Damla Günenç Eşim, Halil Taşkaynatan, Özlem Özdemir

**Affiliations:** 1 Department of Medical Oncology, Izmir City Hospital, Izmir 35540, Turkey; gurkangl6290@gmail.com (G.G.); d.gunenc@yahoo.com.tr (D.G.E.); haliltaskaynatan@gmail.com (H.T.); ozdemirozlem.md@gmail.com (Ö.Ö.); 2 Department of Pathology, Izmir City Hospital, Izmir 35540, Turkey; simgeerbil@yahoo.com (S.B.); asumanargon@gmail.com (A.A.)

**Keywords:** gastric cancer, immunoscore, neoadjuvant chemotherapy, tumor immune microenvironment, pathological response, disease-free survival

## Abstract

**Background/Objectives:** Pathological response after neoadjuvant chemotherapy is an established prognostic factor in locally advanced gastric cancer, but it does not fully reflect the biological heterogeneity of residual disease. The immune microenvironment may provide additional prognostic information. We therefore investigated the relationship between post-neoadjuvant Immunoscore, pathological findings, and disease-free survival in patients with microsatellite-stable locally advanced gastric cancer. **Methods:** This retrospective, single-center study included patients with locally advanced gastric adenocarcinoma who received four cycles of neoadjuvant FLOT followed by curative-intent gastrectomy between November 2023 and May 2025. Immunoscore was determined in post-neoadjuvant resection specimens based on CD3^+^ and CD8^+^ T-cell infiltration in the tumor center and invasive margin. Immunoscore assessments were performed by two pathologists blinded to clinical and outcome data, with final evaluations established through consensus review. Associations between Immunoscore and clinicopathological features were examined, including exploratory multivariable analyses adjusting for baseline clinical T stage and histological subtype. Disease-free survival was analyzed using the Kaplan–Meier method and Cox proportional hazards regression. **Results:** The final analysis included 48 patients. Compared with the low-Immunoscore group, the high-Immunoscore group had lower rates of ypT4 disease (37.5% vs. 75.0%; *p* = 0.014), ypN3 disease (21.9% vs. 68.8%; *p* = 0.002), and perineural invasion (43.8% vs. 75.0%; *p* = 0.041). The association between low Immunoscore and ypN3 disease persisted after adjustment for baseline clinical T stage and histological subtype (adjusted OR 5.89, 95% CI 1.32–26.23; *p* = 0.020). Tumor regression grade did not differ significantly between the groups (*p* = 0.211). During a median follow-up of 18.0 months, 17 patients developed recurrence. Disease-free survival was numerically longer in the high-Immunoscore group. The difference was not significant by the log-rank test (*p* = 0.100), whereas an exploratory Breslow analysis showed a difference between the survival distributions (*p* = 0.025). However, Cox regression did not demonstrate a statistically significant association with disease-free survival (HR 0.47, 95% CI 0.18–1.21; *p* = 0.117). **Conclusions:** A higher post-neoadjuvant Immunoscore was associated with less advanced residual tumor and nodal disease and less frequent perineural invasion, without a statistically significant association with tumor regression grade or disease-free survival in the primary analyses. Immunoscore may therefore complement conventional pathological assessment after neoadjuvant FLOT. These findings require validation in larger prospective cohorts.

## Simple Summary

Patients with locally advanced gastric cancer can have markedly different outcomes even when they receive similar treatments and have comparable disease stages. This highlights the need for additional markers that better reflect tumor biology and help identify patients at higher risk of recurrence. The Immunoscore measures the immune response within and around the tumor by assessing specific types of tumor-infiltrating immune cells. In this study, we evaluated the Immunoscore in surgical specimens from patients with microsatellite-stable locally advanced gastric cancer who had received chemotherapy before surgery. A higher Immunoscore was associated with less advanced residual tumor and nodal disease and less frequent perineural invasion, but it was not significantly associated with conventional tumor regression grade. Although patients with higher Immunoscores had numerically longer disease-free survival, this association was not statistically significant in the primary survival analysis. These findings suggest that the Immunoscore may complement conventional pathological assessment and warrant further investigation as a potential marker for postoperative risk stratification after neoadjuvant treatment.

## 1. Introduction

Gastric cancer remains the fifth leading cause of cancer-related mortality worldwide [1]. A substantial proportion of patients are diagnosed with advanced disease, which is associated with poor clinical outcomes. For locally advanced gastric cancer (LAGC), the standard treatment approach includes perioperative systemic therapy followed by surgical resection. Although this multimodal strategy has improved treatment outcomes, long-term survival remains suboptimal, and considerable variation in clinical outcomes is observed among patients.

Pathological response to neoadjuvant chemotherapy is an established prognostic factor in LAGC; however, its prognostic value varies across studies and patient populations [2,3]. Patients with a poor pathological response generally have worse survival outcomes, and the optimal management of this subgroup remains a clinical challenge [4]. Given the biological and clinical heterogeneity of gastric cancer, patients with similar American Joint Committee on Cancer (AJCC) tumor–node–metastasis (TNM) stages may experience markedly different disease courses, underscoring the limitations of conventional staging in predicting individual outcomes [5,6]. The eighth edition of the AJCC TNM classification was used in the present study to define clinical disease stage and assess post-neoadjuvant pathological findings. Beyond TNM stage, established clinicopathological prognostic factors include histological subtype, tumor differentiation, lymphovascular and perineural invasion, resection margin status, and pathological response to neoadjuvant treatment. Molecular characteristics, including microsatellite instability and Epstein–Barr virus-associated tumor biology, may also influence prognosis and treatment response. Nevertheless, these factors do not fully account for the heterogeneity of clinical outcomes [7].

Increasing attention has therefore been directed toward the tumor microenvironment, a complex network of immune cells, fibroblasts, epithelial cells, and stromal components [8]. This microenvironment includes cytotoxic and helper T lymphocytes, regulatory T cells, tumor-associated macrophages, cancer-associated fibroblasts, and extracellular matrix components, which interact to shape antitumor immunity and tumor progression. CD8^+^ cytotoxic T cells contribute to tumor cell elimination, whereas CD4^+^ T-cell subsets may support or suppress antitumor responses depending on their functional phenotype. Regulatory T cells, often characterized by FOXP3 expression, and immunosuppressive macrophage populations may facilitate immune evasion, while cancer-associated fibroblasts can influence immune cell recruitment and infiltration through stromal remodeling. In particular, the host immune response, reflected by the density and spatial distribution of tumor-infiltrating lymphocytes, plays an important role in tumor progression, treatment response, and clinical outcomes [9]. Higher levels of immune-cell infiltration, particularly by CD3^+^ and CD8^+^ T cells, have been associated with improved survival in gastric cancer [10]. However, the prognostic implications of immune infiltration depend not only on lymphocyte density but also on immune cell phenotype, spatial distribution, and interactions with stromal and immunosuppressive components.

The Immunoscore is a standardized scoring system based on the density and spatial distribution of CD3^+^ and CD8^+^ T cells within the tumor center and invasive margin. It was initially developed and subsequently validated as a prognostic marker in colorectal cancer (CRC) [11,12]. The term “Immunoscore” is therefore well established in the literature, although the internationally validated colorectal cancer assay should be distinguished from adapted immune scoring approaches used in other tumor types.

Although immune-based scoring approaches have also shown prognostic value in gastric cancer, their clinical application remains limited and requires further validation [13]. In particular, differences in scoring methods and cutoff values have limited the establishment of a universally accepted Immunoscore system for gastric cancer. Other relevant microenvironmental markers, including FOXP3, programmed cell death protein 1 (PD-1), and alpha-smooth muscle actin (α-SMA), provide complementary information on regulatory T-cell infiltration, immune checkpoint signaling, and stromal fibroblast activation, respectively. However, the present study focused on CD3^+^ and CD8^+^ T-cell infiltration to evaluate a predefined, Immunoscore-based approach rather than undertake comprehensive immune and stromal profiling. Moreover, most available studies have evaluated pretreatment biopsy specimens or heterogeneous patient populations, whereas data on Immunoscore assessment in post-neoadjuvant resection specimens remain scarce.

With the emergence of neoadjuvant chemo-immunotherapy strategies, including perioperative chemotherapy combined with immune checkpoint inhibitors such as durvalumab, reliable immune-based biomarkers are likely to become increasingly relevant to treatment personalization. However, the clinical significance of Immunoscore in patients treated with neoadjuvant chemotherapy alone, particularly when assessed in surgical resection specimens, remains poorly defined.

In this study, we investigated the association between Immunoscore assessed in post-neoadjuvant resection specimens and pathological features and clinical outcomes in patients with LAGC. By integrating Immunoscore with conventional pathological assessment, we sought to determine whether post-treatment immune contexture could provide additional information for risk stratification and response assessment after neoadjuvant chemotherapy.

## 2. Materials and Methods

### 2.1. Study Design and Patient Selection

This retrospective, single-center cohort study included patients with locally advanced gastric adenocarcinoma treated at Izmir City Hospital between November 2023 and May 2025.

Eligible patients had histologically confirmed gastric adenocarcinoma classified as locally advanced disease (clinical stage ≥ T2 and/or node-positive) according to the 8th edition of the American Joint Committee on Cancer (AJCC) TNM classification. All patients received four cycles of neoadjuvant FLOT chemotherapy followed by curative-intent gastrectomy. Only patients with surgical resection specimens available in the institutional pathology archive were included. Patients with microsatellite instability-high/mismatch repair-deficient (MSI-H/dMMR) tumors were excluded by design to minimize biological and treatment-related heterogeneity, given the distinct tumor immune microenvironment and potentially different therapeutic approaches associated with this molecular subgroup. Accordingly, the study population was restricted to patients with microsatellite-stable (MSS) tumors. Patients with distant metastases at diagnosis, a history of prior malignancy, or incomplete clinicopathological data were excluded.

A total of 51 patients were initially identified as potentially eligible. Three patients had a pathological complete response (tumor regression grade 0), with no residual tumor identified in the resection specimen. Because Immunoscore assessment required residual tumor tissue, these patients were excluded, leaving 48 patients for the final analysis.

### 2.2. Pathological Evaluation and Tumor Regression Grading

All resection specimens were processed according to standard institutional pathology protocols, and pathological staging was performed according to the AJCC 8th edition TNM classification.

Histopathological response to neoadjuvant chemotherapy was assessed on hematoxylin and eosin (H&E)-stained sections using the Ryan tumor regression grading system [14]. Tumor regression was categorized as grade 0 (complete response, no viable tumor cells), grade 1 (near-complete response with minimal residual tumor), grade 2 (partial response with evident residual tumor), or grade 3 (poor or no response with extensive residual tumor).

Post-treatment pathological parameters, including ypT stage, ypN stage, lymphovascular invasion, and perineural invasion, were systematically recorded.

Pathological assessments were jointly performed by two experienced gastrointestinal pathologists (A.A. and S.B.) who were blinded to the patients’ clinical and outcome data. Final pathological assessments were established by consensus.

Microsatellite status was assessed by immunohistochemistry for the mismatch repair proteins MLH1, PMS2, MSH2, and MSH6. Cases with inadequate or equivocal immunohistochemical staining underwent confirmatory PCR-based testing to establish microsatellite status.

### 2.3. Immunohistochemistry and Immunoscore Assessment

Representative formalin-fixed, paraffin-embedded tissue blocks showing the most prominent lymphocytic infiltration were selected for each case. Section 3 and Section 4 μm thick were prepared and subjected to immunohistochemical staining with anti-CD3 (clone 2GV6, Roche, Ready-to-Use, catalog no. 790-4341) and anti-CD8 (clone SP57, Roche, Ready-to-Use, catalog no. 593-7248) antibodies using an automated Ventana Benchmark Ultra staining platform.

CD3^+^ and CD8^+^ T lymphocytes were evaluated in both the tumor center and invasive margin. Manual counting was used rather than automated image analysis to facilitate the exclusion of necrotic areas and accurate identification of the relevant tumor regions. In post-neoadjuvant resection specimens, areas showing the most prominent immune cell infiltration (hotspots) within the residual tumor center and invasive margin were selected for assessment. The assessments were performed jointly by two pathologists blinded to clinical and outcome data, and a single consensus Immunoscore was assigned to each case. As separate observer-specific scores were not recorded, interobserver agreement could not be formally assessed.

Immunoscore was assessed using an adapted four-component scoring approach based on the Immunoscore framework described by Galon and colleagues [15]. The four components (CD3-CT, CD3-IM, CD8-CT, and CD8-IM) were each assigned a score of 0 (low infiltration) or 1 (high infiltration), yielding a total Immunoscore ranging from 0 to 4. For the present exploratory analysis, patients were categorized into low Immunoscore (0–1) and high Immunoscore (2–4) groups. This exploratory dichotomization was intended to distinguish tumors with relatively limited immune infiltration from those with greater infiltration while avoiding small subgroups. Although conceptually consistent with previous Immunoscore studies comparing low with combined intermediate and high categories, this cutoff has not been independently validated in gastric cancer and differs from the standardized colorectal Consensus Immunoscore classification.

Representative histopathological and immunohistochemical findings from tumors with low and high Immunoscores are shown in Figure 1 and Figure 2, respectively.

### 2.4. Data Collection and Follow-Up

Clinical and pathological data, including age, sex, tumor location, histological subtype, clinical stage, treatment details, and pathological response, were retrieved from electronic medical records. Follow-up information was obtained from outpatient records and hospital databases, with a data cutoff in February 2026.

Disease-free survival (DFS) was defined as the time from surgery to the first documented recurrence, as determined by radiological imaging. Patients without recurrence were censored at the date of their last disease-free follow-up.

### 2.5. Statistical Analysis

Statistical analyses were performed using SPSS version 26.0 (IBM Corp., Armonk, NY, USA).

The distribution of continuous variables was assessed visually using histograms and Q–Q plots and analytically using the Kolmogorov–Smirnov or Shapiro–Wilk test. Parametric tests were used for variables with an approximately normal distribution.

Comparisons between the low- and high-Immunoscore groups were performed using independent-samples t-tests for continuous variables and the chi-square or Fisher’s exact test for categorical variables, as appropriate.

An exploratory multivariable binary logistic regression analysis was performed to assess the association between Immunoscore and ypN3 disease after adjustment for baseline clinical T stage and histological subtype. Adjusted odds ratios (ORs) and 95% confidence intervals (CIs) were reported. To assess the robustness of the findings to the selected dichotomization threshold, additional logistic regression analyses were performed using the original Immunoscore (0–4) as an ordinal predictor, modeled per one-point increase, both before and after adjustment for baseline clinical T stage and histological subtype. The association between ordinal Immunoscore and tumor regression grade (TRG) was further examined using Spearman’s rank correlation coefficient.

Disease-free survival was estimated using the Kaplan–Meier method. Survival curves were compared primarily using the log-rank test. The Breslow (generalized Wilcoxon) test was additionally used to examine differences occurring earlier during follow-up. The Breslow test was considered exploratory, and its findings were interpreted in the context of the primary log-rank analysis. Cox proportional hazards regression was used to estimate hazard ratios (HRs) and 95% CIs for disease-free survival. Additional sensitivity analyses examined Immunoscore as an ordinal predictor (0–4), including a model adjusted for baseline clinical T stage.

Given the limited sample size and number of events, multivariable analyses were considered exploratory, and the number of covariates was restricted to reduce the risk of overfitting.

All statistical tests were two-sided, and a *p*-value < 0.05 was considered statistically significant.

## 3. Results

### 3.1. Baseline Clinicopathological Characteristics According to Immunoscore Status

A total of 48 patients were included in the final analysis, of whom 16 had a low Immunoscore and 32 had a high Immunoscore. Baseline clinicopathological characteristics according to Immunoscore status are presented in Table 1.

Patients in the high-Immunoscore group were more likely to have conventional adenocarcinoma histology than those in the low-Immunoscore group (84.4% vs. 50.0%, *p* = 0.041). The proportion of patients with cT4 disease was also lower in the high-Immunoscore group (15.6% vs. 50.0%, *p* = 0.018). The distribution of overall clinical stage differed between the groups (*p* = 0.044), with stage II–IIIA disease being more frequent in the high-Immunoscore group (90.6% vs. 62.5%). No significant differences were observed between the groups in age, sex, or clinical nodal stage.

### 3.2. Post-Neoadjuvant Pathological Characteristics According to Immunoscore Status

Post-neoadjuvant pathological findings according to Immunoscore status are presented in Table 2.

Although Ryan grade 1 regression was numerically more frequent in the high-Immunoscore group than in the low-Immunoscore group (34.4% vs. 12.5%), the overall distribution of TRG categories did not differ significantly between groups (*p* = 0.211). An additional analysis using the original 0–4 Immunoscore showed no statistically significant monotonic association with TRG (Spearman’s ρ = −0.148, *p* = 0.314). The distribution of TRG across individual Immunoscore categories (0–4) is shown in Figure 3.

In contrast, post-treatment tumor and nodal stages differed between the groups. Patients in the high-Immunoscore group had lower rates of ypT4 disease (37.5% vs. 75.0%, *p* = 0.014) and ypN3 disease (21.9% vs. 68.8%, *p* = 0.002) than those in the low-Immunoscore group. Perineural invasion was also less frequent in the high-Immunoscore group (43.8% vs. 75.0%, *p* = 0.041), whereas the frequency of lymphovascular invasion did not differ significantly between the groups (53.1% vs. 75.0%, *p* = 0.144).

In an exploratory multivariable logistic regression analysis adjusting for baseline clinical T stage and histological subtype, low Immunoscore was associated with higher odds of ypN3 disease compared with high Immunoscore (adjusted OR 5.89, 95% CI 1.32–26.23; *p* = 0.020). In a sensitivity analysis treating Immunoscore as an ordinal variable (0–4), each one-point increase was associated with lower odds of ypN3 disease after adjustment for baseline clinical T stage and histological subtype (adjusted OR 0.38, 95% CI 0.19–0.77; *p* = 0.007).

### 3.3. Clinical-to-Pathological Stage Transitions According to Immunoscore Status

Transitions from pretreatment clinical T and N categories to post-neoadjuvant pathological T and N categories were examined descriptively according to Immunoscore status. Patient-level transitions from cT to ypT and from cN to ypN are illustrated using Sankey diagrams (Figure 4).

### 3.4. Survival Analysis

The median follow-up duration was 18.0 months (95% CI, 15.9–20.1), during which 17 patients (35.4%) experienced disease recurrence. Kaplan–Meier curves showed an early separation in disease-free survival between the high- and low-Immunoscore groups (Figure 5).

Mean disease-free survival was 19.0 months (95% CI, 13.3–24.7) in the low-Immunoscore group and 22.6 months (95% CI, 19.8–25.4) in the high-Immunoscore group. Median disease-free survival was 17.0 months (95% CI, 5.5–28.5) and 25.0 months (95% CI, 21.3–28.7), respectively.

The difference in disease-free survival between the groups was not statistically significant by the log-rank test (*p* = 0.100). In an exploratory analysis giving greater weight to earlier events, the Breslow test showed a difference between the survival distributions (*p* = 0.025).

In univariable Cox proportional hazards analysis, high Immunoscore was associated with a numerically lower hazard of DFS events compared with low Immunoscore, although this association did not reach statistical significance (HR 0.47, 95% CI 0.18–1.21; *p* = 0.117).

Similarly, when Immunoscore was analyzed as an ordinal variable, each one-point increase was associated with a numerically lower hazard of DFS events, although the association was not statistically significant (HR 0.73, 95% CI 0.50–1.08; *p* = 0.115).

In an exploratory Cox regression analysis adjusted for baseline clinical T stage, the association remained statistically nonsignificant (adjusted HR 0.80 per one-point increase in Immunoscore, 95% CI 0.54–1.19; *p* = 0.270).

## 4. Discussion

In this study, we evaluated the clinicopathological significance of Immunoscore assessed in post-neoadjuvant resection specimens from patients with microsatellite-stable locally advanced gastric cancer who received four cycles of neoadjuvant FLOT. Immunoscore was not significantly associated with tumor regression grade. However, patients with a high Immunoscore had lower rates of ypT4 and ypN3 disease and perineural invasion. Disease-free survival was numerically longer in the high-Immunoscore group, although the difference was not significant when assessed by the log-rank test. Taken together, these findings suggest that post-treatment immune contexture may provide information on residual disease characteristics that is not fully captured by tumor regression grade.

Tumor regression grading reflects the extent of residual viable tumor following neoadjuvant therapy [16]. Its assessment in gastric cancer, however, is complicated by the use of multiple grading systems with different definitions and thresholds [17], as well as reported interobserver variability [18]. These factors may contribute to variation in the reproducibility and prognostic performance of tumor regression grading. Given the role of the tumor immune microenvironment in gastric cancer progression and prognosis, immune-based measures may complement morphology-based assessment of treatment response [19].

Our finding that Immunoscore was associated with several post-treatment pathological features but not with tumor regression grade is noteworthy. Tumor regression grading primarily describes morphological evidence of treatment response, whereas it does not directly capture host immune activity or treatment-related changes in the tumor microenvironment [20,21]. Immunoscore, by contrast, incorporates the density and spatial distribution of CD3^+^ and CD8^+^ T cells within the tumor and its invasive margin [22]. The two measures may therefore reflect different aspects of the residual tumor after neoadjuvant treatment rather than providing interchangeable information. However, the absence of a statistically significant association between Immunoscore and TRG should not be interpreted as evidence of biological independence. Although favorable tumor regression was numerically more frequent in patients with higher Immunoscores, the modest sample size and limited numbers within individual TRG categories may have reduced the ability to detect an association. Differences between the findings for ypT/ypN stage and TRG may therefore reflect statistical limitations rather than distinct biological relationships.

The lower frequency of ypN3 disease in patients with a high Immunoscore is also consistent with a potential relationship between an immune-active tumor microenvironment and a lower residual nodal burden. Previous studies in gastric cancer have associated greater CD3^+^ and CD8^+^ T-cell infiltration with favorable pathological characteristics and survival outcomes, including in microsatellite-stable disease [23]. Nevertheless, this association should be interpreted cautiously in our cohort. The Immunoscore groups differed in several baseline characteristics, including clinical T stage, overall clinical stage, and histological subtype. The association between low Immunoscore and ypN3 disease persisted after adjustment for baseline clinical T stage and histological subtype. Nevertheless, the small sample size and wide confidence interval limit the precision of this estimate, and residual confounding cannot be excluded. Accordingly, these findings support an association between post-treatment Immunoscore and residual nodal disease but do not establish an independent biological or causal effect of Immunoscore.

Sensitivity analyses using the original 0–4 Immunoscore yielded findings consistent with those obtained using the dichotomized classification. The association with advanced pathological nodal involvement persisted, whereas the association with DFS remained statistically nonsignificant. Nevertheless, the selected dichotomization threshold has not been independently validated in gastric cancer, and the present findings should be considered exploratory.

Evidence on Immunoscore assessment specifically in post-neoadjuvant gastrectomy specimens remains limited, and relatively few studies have examined immune contexture after systemic therapy [24]. Our study adds to this literature by evaluating a homogeneous cohort of patients with microsatellite-stable disease treated with the same neoadjuvant regimen. The associations of higher Immunoscore with lower ypT and ypN categories and less frequent perineural invasion suggest that immune assessment of the resection specimen may provide a different perspective on residual disease after FLOT. Whether these findings reflect pre-existing tumor immune biology, treatment-induced immune remodeling, or both cannot be determined from post-treatment specimens alone.

In the survival analysis, patients with a high Immunoscore had numerically longer disease-free survival than those with a low Immunoscore. The difference was not statistically significant when assessed by the log-rank test (*p* = 0.100), which should be considered the primary survival comparison. An exploratory Breslow analysis, which gives greater weight to earlier events, showed a difference between the survival distributions (*p* = 0.025). Although this finding is consistent with the early separation of the Kaplan–Meier curves, it should not be interpreted as definitive evidence of a survival benefit. Neither the univariable Cox regression analyses nor the exploratory Cox model adjusted for baseline clinical T stage demonstrated a statistically significant association between Immunoscore and DFS. The relatively short follow-up and limited number of recurrence events restrict the reliability of long-term survival estimates and may also influence the apparent early separation of the curves. Thus, the isolated Breslow finding should be interpreted cautiously, and no definitive conclusion regarding the prognostic value of Immunoscore for DFS can be drawn from this cohort.

These findings may have particular relevance as perioperative treatment for locally advanced gastric cancer increasingly incorporates immune-based strategies [25]. Post-treatment immune assessment could potentially complement conventional pathological variables when characterizing residual disease, but the present data are not sufficient to support its use for treatment decisions. Future studies should determine whether Immunoscore adds prognostic information beyond established factors such as ypT stage, ypN stage, histological response, and other clinicopathological characteristics. Evaluation in larger cohorts will also be necessary to establish clinically meaningful thresholds and determine whether Immunoscore can contribute to postoperative risk stratification.

Assessment of immune contexture in pretreatment endoscopic biopsies represents another relevant direction for future research. Although Immunoscore assessment incorporates immune infiltration in both the tumor center and invasive margin, which can be more comprehensively evaluated in surgical resection specimens, neoadjuvant chemotherapy may alter the tumor immune microenvironment. In the present study, restricting the cohort to patients with microsatellite-stable disease treated with a uniform neoadjuvant FLOT regimen was intended to reduce biological and treatment-related heterogeneity, but this approach cannot account for individual treatment-induced immune changes. Although biopsy specimens provide limited tissue and are susceptible to sampling error and intratumoral heterogeneity, paired evaluation of pretreatment biopsies and post-treatment resection specimens could help distinguish baseline immune characteristics from changes associated with neoadjuvant therapy. Such studies could also clarify whether dynamic changes in Immunoscore are more informative than a single post-treatment measurement and whether immune-based measures can contribute to treatment selection before surgery.

This study has several limitations. The small sample size and limited number of recurrence events (*n* = 17) may have reduced the precision of survival estimates and limited the ability to detect clinically meaningful differences in DFS. Although exploratory multivariable analyses were performed, the baseline differences between the low- and high-Immunoscore groups could not be fully accounted for, and the limited number of events restricted the complexity of the survival models. In addition, Immunoscore was assessed only in post-neoadjuvant resection specimens, precluding evaluation of treatment-related changes in immune contexture. The relatively short follow-up further limits interpretation of the survival findings. Moreover, the multiple clinicopathological comparisons increase the possibility of chance findings. Therefore, the observed associations should be interpreted as exploratory and hypothesis-generating rather than definitive evidence of prognostic significance.

Although pathological and Immunoscore assessments were finalized through consensus review by two pathologists, individual pre-consensus scores were not systematically recorded. Consequently, interobserver agreement could not be formally assessed, limiting the evaluation of the reproducibility of the scoring method.

In addition, although the final consensus Immunoscore was available for each patient, the original component-level cell counts and numerical thresholds used to distinguish low from high infiltration were not retained in the clinical research dataset. Consequently, the original scoring thresholds could not be retrospectively verified, limiting the reproducibility of the adapted scoring approach.

The predefined exclusion of MSI-H/dMMR tumors limits the generalizability of our findings, particularly given the distinct immune microenvironment and evolving treatment strategies in this subgroup. Therefore, our results should be interpreted within the context of MSS locally advanced gastric cancer treated with neoadjuvant FLOT followed by surgery.

Strengths of the study include the homogeneous microsatellite-stable population, use of a uniform neoadjuvant FLOT regimen, and consensus-based pathological assessment by two pathologists blinded to clinical and outcome data. Larger prospective multicenter studies incorporating paired pretreatment and post-treatment specimens are needed to validate these findings and clarify the prognostic value of Immunoscore after neoadjuvant therapy.

## 5. Conclusions

In patients with microsatellite-stable locally advanced gastric cancer treated with neoadjuvant FLOT, a higher post-neoadjuvant Immunoscore was associated with less advanced residual tumor and nodal disease and less frequent perineural invasion, whereas no statistically significant association was observed with tumor regression grade.

These findings suggest that Immunoscore may capture features of residual disease biology that are not fully reflected by conventional morphological response assessment, although the absence of a significant association with tumor regression grade does not establish biological independence.

Although disease-free survival was numerically longer in patients with a higher Immunoscore, neither the log-rank test nor Cox regression analyses demonstrated a statistically significant association. The survival findings remain exploratory and require validation in larger cohorts with longer follow-up. Prospective studies incorporating both pretreatment and post-treatment specimens are needed to determine whether Immunoscore provides prognostic information beyond established clinicopathological factors and whether it can contribute to risk stratification after neoadjuvant treatment.

## Figures and Tables

**Figure 1:**
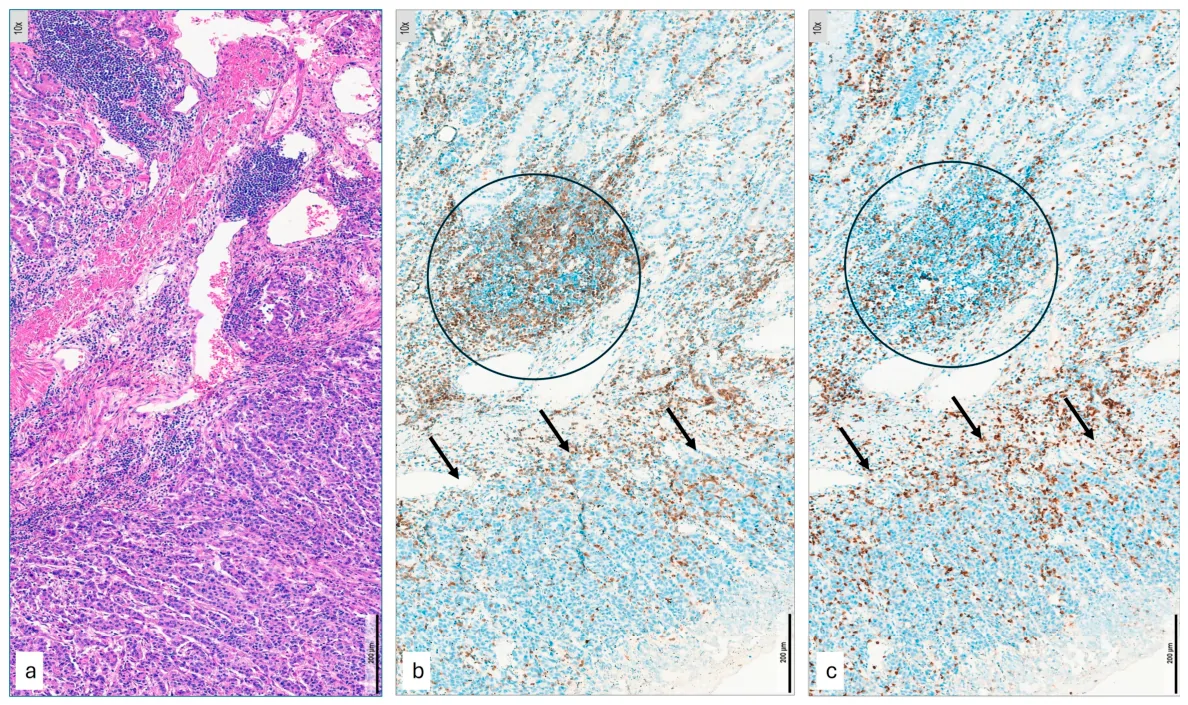
Representative histopathological and immunohistochemical findings in a tumor with an Immunoscore of 1. (**a**) H&E-stained section showing normal gastric mucosa and mucosa-associated lymphoid tissue in the upper part and residual poorly differentiated adenocarcinoma in the lower part of the image (×10). (**b**) CD3 immunohistochemistry showing sparse CD3^+^ T-cell infiltration within the residual tumor. The circled area indicates mucosa-associated lymphoid tissue serving as an internal positive control, whereas the black arrows indicate the tumor area (×10). (**c**) CD8 immunohistochemistry showing CD8^+^ T-cell infiltration within the residual tumor. The circled area indicates mucosa-associated lymphoid tissue serving as an internal positive control, whereas the black arrows indicate the tumor area (×10). Scale bars, 200 μm.

**Figure 2:**
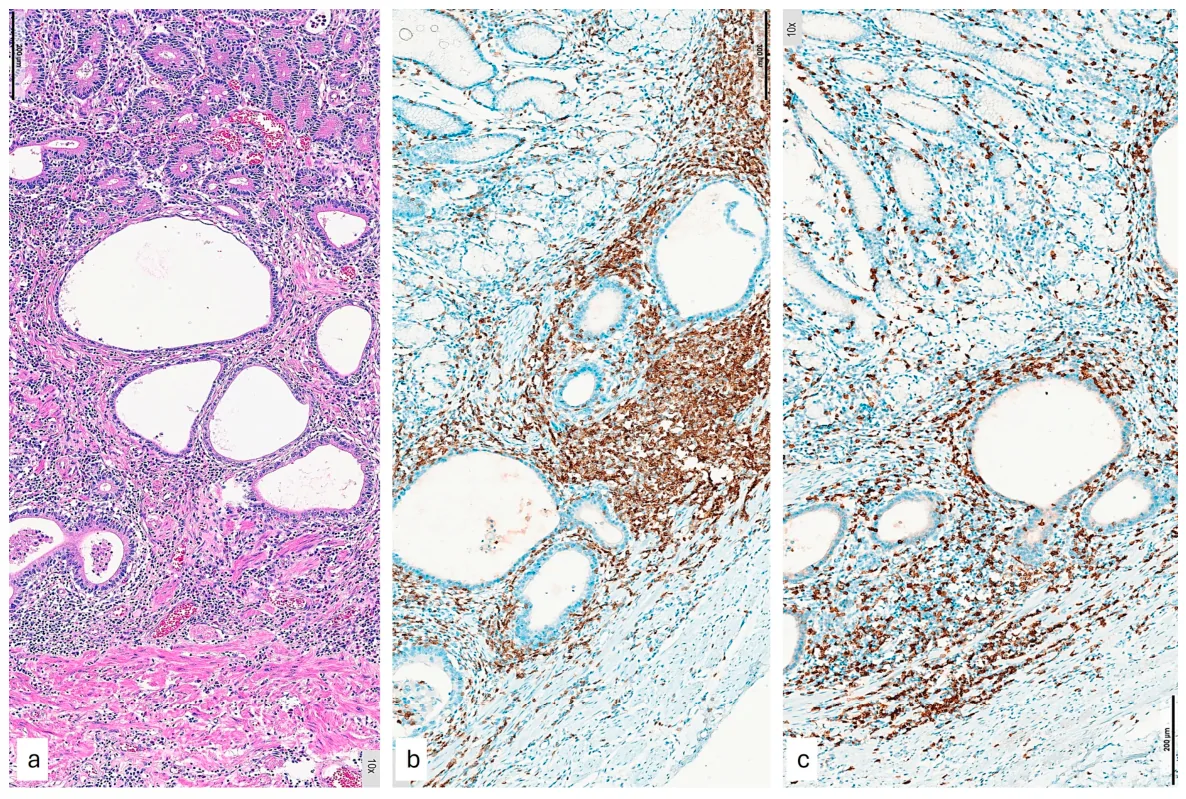
Representative histopathological and immunohistochemical findings in a tumor with an Immunoscore of 4. (**a**) H&E-stained section showing normal gastric mucosa in the upper part and residual well-differentiated adenocarcinoma in the submucosa (×10). (**b**) CD3 immunohistochemistry showing abundant CD3^+^ T-cell infiltration within and around the residual tumor (×10). (**c**) CD8 immunohistochemistry showing dense CD8^+^ T-cell infiltration within and around the residual tumor (×10). Scale bars, 200 μm.

**Figure 3:**
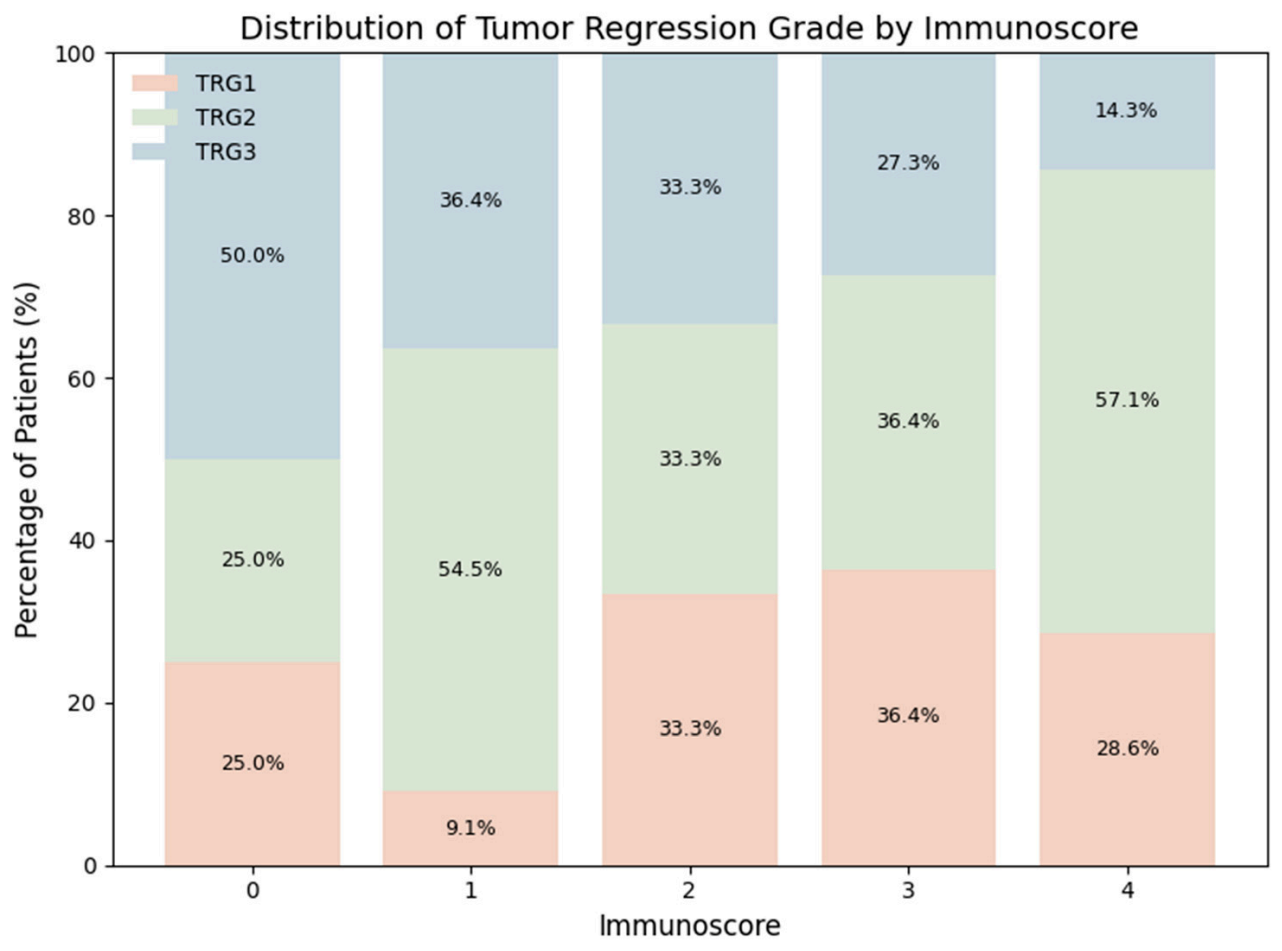
Distribution of tumor regression grade according to Immunoscore. Stacked bars show the proportions of patients with Ryan tumor regression grades 1, 2, and 3 across individual Immunoscore categories (0–4). TRG, tumor regression grade.

**Figure 4:**
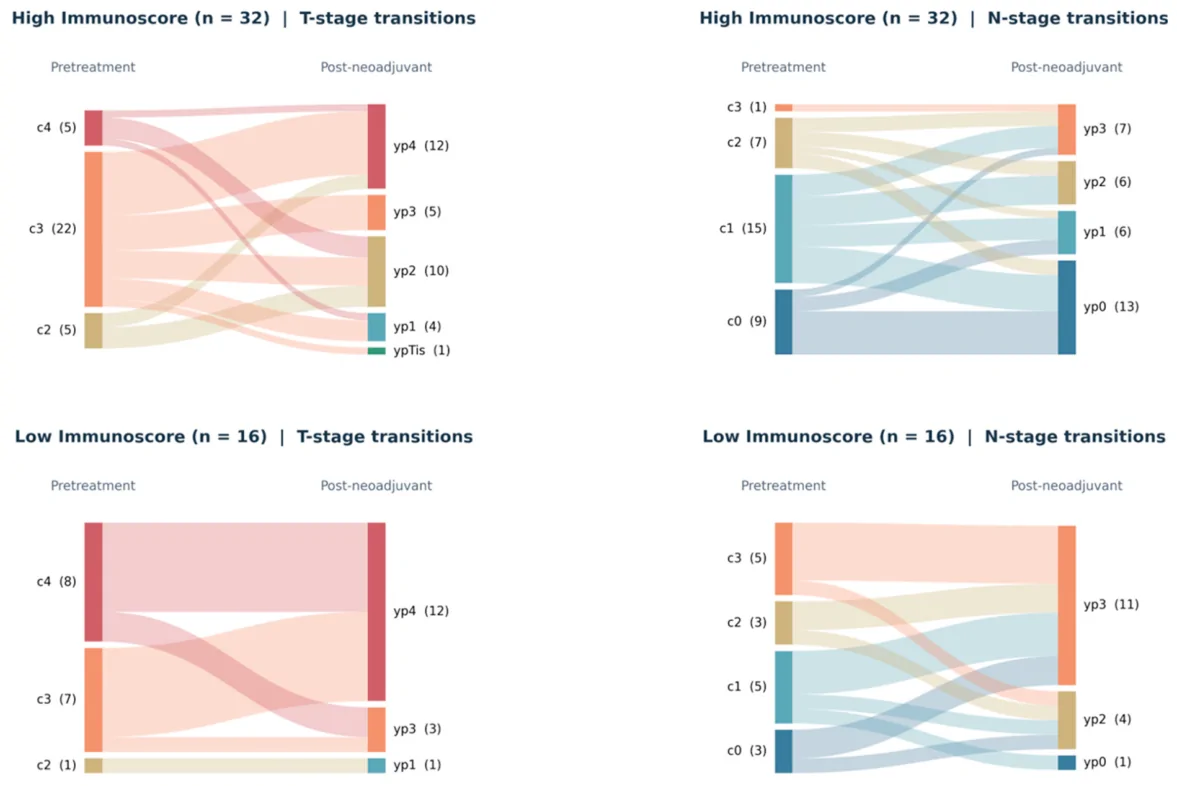
Clinical-to-pathological T- and N-stage transitions according to Immunoscore status. Sankey diagrams illustrate patient-level transitions from pretreatment clinical T (cT) and N (cN) categories to post-neoadjuvant pathological T (ypT) and N (ypN) categories in patients with high (*n* = 32) and low (*n* = 16) Immunoscores. Flow widths are proportional to patient counts. Pathological N3a and N3b categories were combined as ypN3. Advanced residual pathological disease, particularly ypT4 and ypN3, was more frequent in the low-Immunoscore group. These transitions are descriptive and should not be interpreted as evidence of treatment-induced stage progression or differences in downstaging between groups.

**Figure 5:**
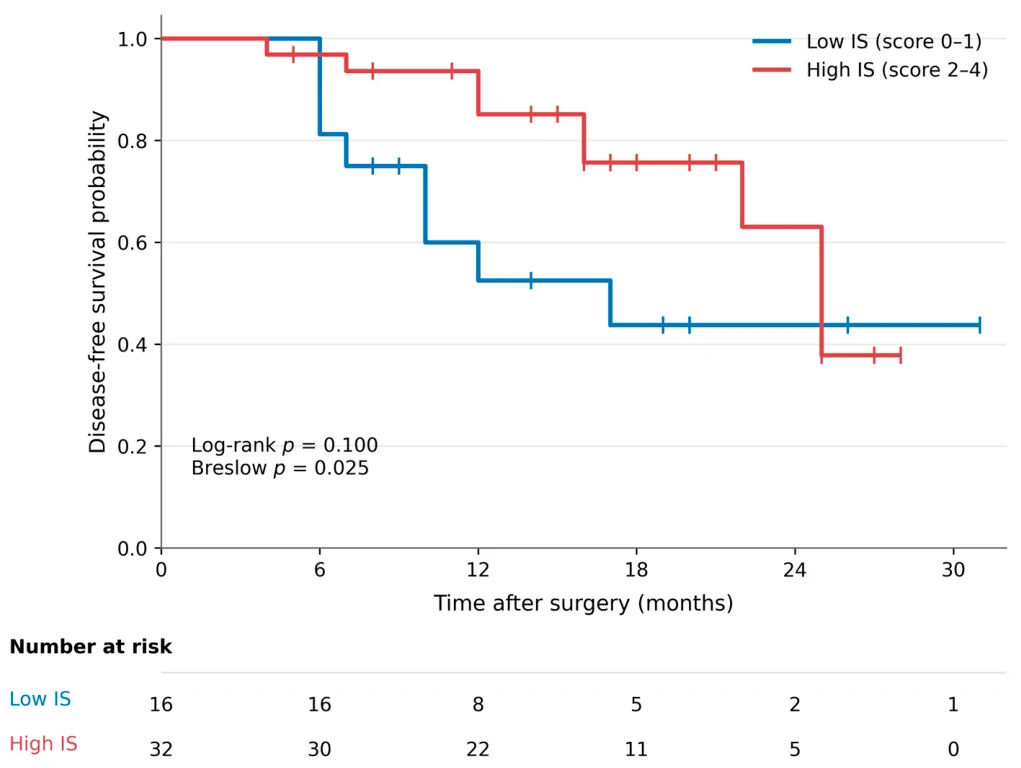
Kaplan–Meier analysis of disease-free survival according to Immunoscore status. Disease-free survival was compared between patients with low Immunoscore (scores 0–1) and high Immunoscore (scores 2–4). The difference between the groups was not statistically significant by the log-rank test (*p* = 0.100). An exploratory Breslow (generalized Wilcoxon) test, which gave greater weight to earlier events, yielded a nominally significant difference (*p* = 0.025). DFS, disease-free survival.

**Table 1:** Baseline clinicopathological characteristics according to Immunoscore status.

Variable	All (*n* = 48)	Low IS (*n* = 16)	High IS (*n* = 32)	*p* Value
**Age (years)**				0.838
<65	25 (52.1%)	8 (50%)	17 (53.1%)	
≥65	23 (47.9%)	8 (50%)	15 (46.9%)	
**Sex**				0.058
Male	30 (62.5%)	7 (43.8%)	23 (71.9%)	
Female	18 (37.5%)	9 (56.2%)	9 (28.1%)	
**Histology**				0.041
Adenocarcinoma	35 (72.9%)	8 (50%)	27 (84.4%)	
Signet ring cell	8 (16.7%)	5 (31.3%)	3 (9.4%)	
Other	5 (10.4%)	3 (18.7%)	2 (6.2%)	
**Signet ring cell component**				0.095
Present	19 (39.6%)	9 (56.3)	10 (31.3%)	
Absent	29 (60.4%)	7 (43.8)	22 (68.8%)	
**Clinical T stage** *				0.018
cT2–3	35 (72.9%)	8 (50%)	27 (84.4%)	
cT4	13 (27.1)	8 (50%)	5 (15.6%)	
**Clinical N stage**				0.083
cN0–1	32 (66.7%)	8 (50%)	24 (75%)	
cN2–3	16 (33.3%)	8 (50%)	8 (25%)	
**Clinical stage**				0.044
Stage II–IIIA	39 (81.2%)	10 (62.5%)	29 (90.6%)	
Stage IIIB	9 (18.8%)	6 (37.5%)	3 (9.4%)	

Data are presented as *n* (%), with percentages calculated using the total number of patients in each group (low Immunoscore, *n* = 16; high Immunoscore, *n* = 32). IS, Immunoscore; cT, clinical tumor stage; cN, clinical nodal stage. * Fisher’s exact test was used for the comparison of clinical T stage; Pearson’s chi-square test was used for the remaining categorical comparisons.

**Table 2:** Post-neoadjuvant pathological characteristics according to Immunoscore status.

Variable	Low IS (*n* = 16)	High IS (*n* = 32)	*p* Value
**Tumor Regression Grade (Ryan)**			0.211
TRG 1	2 (12.5%)	11 (34.4%)	
TRG 2	7 (43.8%)	13 (40.6%)	
TRG 3	7 (43.8%)	8 (25%)	
**ypT Stage**			0.014
ypT4	12 (75%)	12 (37.5%)	
ypT1-3	4 (25%)	20 (62.5%)	
**ypN Stage**			0.002
ypN3	11 (68.8%)	7 (21.9%)	
ypN0-2	5 (31.2%)	25 (78.1%)	
**Lymphovascular invasion**			0.144
Present	12 (75%)	17 (53.1%)	
Absent	4 (25%)	15 (46.9%)	
**Perineural invasion**			0.041
Present	12 (75%)	14 (43.8%)	
Absent	4 (25%)	18 (56.2%)	

Data are presented as *n* (%), with percentages calculated using the total number of patients in each column as the denominator (low Immunoscore, *n* = 16; high Immunoscore, *n* = 32).

## Data Availability

The data supporting the findings of this study are available from the corresponding author upon reasonable request. The data are not publicly available due to patient privacy and ethical restrictions.

## Abbreviations

The following abbreviations are used in this manuscript:

AJCC	American Joint Committee on Cancer
CD	Cluster of Differentiation
DFS	Disease-Free Survival
FLOT	5-Fluorouracil, Leucovorin, Oxaliplatin, and Docetaxel
H&E	Hematoxylin and Eosin
IS	Immunoscore
LAGC	Locally Advanced Gastric Cancer
MSS	Microsatellite Stable
PNI	Perineural Invasion
LVI	Lymphovascular Invasion
TRG	Tumor Regression Grade
TNM	Tumor–Node–Metastasis
ypN	Post-Neoadjuvant Pathological Nodal Stage
ypT	Post-Neoadjuvant Pathological Tumor Stage

## References

[1] Bray F., Laversanne M., Sung H., Ferlay J., Siegel R.L., Soerjomataram I., Jemal A. (2024). Global cancer statistics 2022: GLOBOCAN estimates of incidence and mortality worldwide for 36 cancers in 185 countries. CA Cancer J. Clin..

[2] Becker K., Mueller J.D., Schulmacher C., Ott K., Fink U., Busch R., Bottcher K., Siewert J.R., Hofler H. (2003). Histomorphology and grading of regression in gastric carcinoma treated with neoadjuvant chemotherapy. Cancer.

[3] Cao Y., Jin T., Chen Z., Du E., Liang P., Gou Y., Yang K. (2026). The association between pathological complete response and prognosis of gastric or adenocarcinoma of esophagogastric junction cancer following neoadjuvant chemotherapy: A meta-analysis. Eur. J. Surg. Oncol..

[4] Derieux S., Svrcek M., Manela S., Lagorce-Pages C., Berger A., André T., Taieb J., Paye F., Voron T. (2020). Evaluation of the prognostic impact of pathologic response to preoperative chemotherapy using Mandard’s tumor regression grade in gastric adenocarcinoma. Dig. Liver Dis..

[5] Dai H., Ren J., Wang C., Huang J., Wang X. (2025). Prognostic molecular subtype reveals the heterogeneity of tumor immune microenvironment in gastric cancer. Sci. Rep..

[6] Grigorie T.R., Verdea C., Chiriac T.D., Gramaticu I.M., Iliesiu A., Popescu G.A., Popescu M., Alexandrescu S.T. (2025). Improving prognostic stratification in gastric cancer: The role of lymph node staging systems. Medicina.

[7] Cancer Genome Atlas Research Network (2014). Comprehensive molecular characterization of gastric adenocarcinoma. Nature.

[8] Yun H., Dong F., Wei X., Yan X., Zhang R., Zhang X., Wang Y. (2024). Role and value of the tumor microenvironment in the progression and treatment resistance of gastric cancer. Oncol. Rep..

[9] Zhang N., Cao M., Duan Y., Bai H., Li X., Wang Y. (2020). Prognostic role of tumor-infiltrating lymphocytes in gastric cancer: A meta-analysis and experimental validation. Arch. Med. Sci..

[10] Lee J.S., Won H.S., Sun D.S., Hong J.H., Ko Y.H. (2018). Prognostic role of tumor-infiltrating lymphocytes in gastric cancer. Medicine.

[11] Galon J., Mlecnik B., Bindea G., Angell H.K., Berger A., Lagorce C., Lugli A., Zlobec I., Hartmann A., Bifulco C. (2014). Towards the introduction of the Immunoscore in the classification of malignant tumours. J. Pathol..

[12] Pagès F., Mlecnik B., Marliot F., Bindea G., Ou F.-S., Bifulco C., Lugli A., Zlobec I., Rau T.T., Berger M.D. (2018). International validation of the consensus Immunoscore for the classification of colon cancer: A prognostic and accuracy study. Lancet.

[13] Jiang Y., Wang H., Wu J., Chen C., Yuan Q., Huang W., Li T., Xi S., Hu Y., Zhou Z. (2020). Noninvasive imaging evaluation of tumor immune microenvironment to predict outcomes in gastric cancer. Ann. Oncol..

[14] Ryan R., Gibbons D., Hyland J.M.P., Treanor D., White A., Mulcahy H.E., O’Donoghue D.P., Moriarty M., Fennelly D., Sheahan K. (2005). Pathological response following long-course neoadjuvant chemoradiotherapy for locally advanced rectal cancer. Histopathology.

[15] Galon J., Pagès F., Marincola F.M., Angell H.K., Thurin M., Lugli A., Zlobec I., Berger A., Bifulco C., Botti G. (2012). Cancer classification using the Immunoscore: A worldwide task force. J. Transl. Med..

[16] Wang Y., Xu H., Hu C., Yu P., Bao Z., Zhang Y., Zhang R., Zhang S., Li B., Aerts J.-M. (2022). Prognostic value and clinicopathological correlation of tumor regression grade after neoadjuvant chemotherapy for gastric adenocarcinoma: A retrospective cohort study. J. Gastrointest. Oncol..

[17] Tsagkalidis V., Blaszczyk M.B., In H. (2023). Interpretation of tumor response grade following preoperative therapy for gastric cancer: An overview. Cancers.

[18] Mirza A., Naveed A., Hayes S., Formela L., Welch I., West C.M., Pritchard S. (2012). Assessment of histopathological response in gastric and gastro-oesophageal junction adenocarcinoma following neoadjuvant chemotherapy: Which scoring system to use?. ISRN Pathol..

[19] Ma M., Sun J., Liu Z., Ouyang S., Zhang Z., Zeng Z., Li J., Kang W. (2022). The immune microenvironment in gastric cancer: Prognostic prediction. Front. Oncol..

[20] Neves Filho E.H.C., de Sant’Ana R.O., Nunes L.V.S.C., Pires A.P.B., da Cunha Mdo P.S.S. (2017). Histopathological regression of gastric adenocarcinoma after neoadjuvant therapy: A critical review. APMIS.

[21] Fang F., Zhang T., Li Q., Chen X., Jiang F., Shen X. (2022). The tumor immune-microenvironment in gastric cancer. Tumori. J..

[22] Zou W., Zhou M., Zhang L., Yang J., Yang W., Wang Y., Yi Y.-X., Li G.-C., Zhang Z. (2021). Immune score predicts outcomes of gastric cancer patients treated with adjuvant chemoradiotherapy. J. Oncol..

[23] Cao X., Kang Y., Tai P., Zhang P., Lin X., Xu F., Nie Z., He B. (2025). Prognostic role of tumor-infiltrating lymphocytes in gastric cancer: A systematic review and meta-analysis. Clin. Res. Hepatol. Gastroenterol..

[24] Zhao S., Liu Y., Ding L., Zhang C., Ye J., Sun K., Song W., Cai S., He Y., Peng J. (2024). Gastric cancer immune microenvironment score predicts neoadjuvant chemotherapy efficacy and prognosis. J. Pathol. Clin. Res..

[25] Janjigian Y.Y., Al-Batran S.E., Wainberg Z.A., Muro K., Molena D., Van Cutsem E., Hyung W.J., Wyrwicz L., Oh D.-Y., Omori T. (2025). Perioperative durvalumab in gastric and gastroesophageal junction cancer. N. Engl. J. Med..

